# Insight into the Mechanisms Underlying the Tracheorelaxant Properties of the *Sideritis raeseri* Extract

**DOI:** 10.1155/2020/6510708

**Published:** 2020-08-28

**Authors:** Berat Krasniqi, Shpëtim Thaçi, Miribane Dërmaku-Sopjani, Arleta Rifati-Nixha, Sokol Abazi, Mentor Sopjani

**Affiliations:** ^1^Faculty of Medicine, University of Prishtina, Prishtina 10 000, Kosovo; ^2^Faculty of Natural Sciences and Mathematics, University of Prishtina, Prishtina 10 000, Kosovo; ^3^Canadian Institute of Technology, Tirana 100, Albania

## Abstract

*Sideritis raeseri* Boiss. and Heldr. (Lamiaceae), known as “mountain tea,” is a native plant from the Mediterranean region, which is widely used in traditional medicine. This study evaluates the effects of the ethanol extract of *Sideritis raeseri* (SR) on airway smooth muscle activity and identifies the underlying mechanism. The *S. raeseri* extract (SRE) was extracted from air-dried parts of the shoot system of SR. The SRE (0.3–2 mg/mL) was tested in isolated rabbit tracheal rings, suspended in the organ bath, filled with Krebs solution, and bubbled with the carbogen mixture (95% O_2_/5% CO_2_) under a resting tension of 1 g in 37°C. In *in vitro* experiments, the SRE relaxed against acetylcholine-induced constriction in tracheal rings. Furthermore, SRE inhibited Ca^2+^-induced contractions in carbachol (CCh, 1 *μ*M) as well as in the K^+^-depolarized trachea (80 mM). Our findings showed the NO/cGMP involvement in tracheorelaxant effects of SR. To this end, the effect of the SRE was potentiated by bradykinin (nitric oxide (NO) synthase activator, 100 nM), whereas it was inhibited by ODQ (inhibitor of NO-sensitive guanylyl cyclase, 10 *μ*M) and L-NAME (NO synthase inhibitor, 100 *μ*M), as well as indomethacin (cyclooxygenase inhibitor, 10 *μ*M). These data suggest that the tracheorelaxant effect of the SRE is mediated at least partly by NO/cyclic guanosine monophosphate and cyclooxygenase-1-prostaglandin E2-dependent signaling. These findings indicate that the SRE may be used in various respiratory disorders.

## 1. Introduction

Medicinal plants have been identified and widely used throughout human history [1]. These plants were important for culinary and particularly to traditional medicine. In many nations, herbal therapies have been used for preventing and treating many diseases, including respiratory diseases [2]. Furthermore, in the last years, numerous natural products have been identified, with strong healing potential in a wide range of human diseases [2–5].

Many plant-derived natural products are approved for therapeutic use in the last decades [6, 7]; however, the underlying mechanisms are poorly understood. Accordingly, the assessment of their pharmacological effects and detailed mechanism of action can further help to advance explaining their functions and can be applied as an attractive strategy for discovering new drugs from plants.

The genus *Sideritis* L. (Lamiaceae) comprises approximately 150 different species. One of them is *Sideritis raeseri* (SR) [5, 7], which is widely distributed in the Mediterranean region (mainly in the Balkans and the Iberian Peninsula) and Macaronesia, but it can also be found in some other places. *S. raeseri* spp. *raeseri* Boiss. and Heldr. (Lamiaceae), known as mountain or shepherd's tea, has been widely used in traditional medicine for its medicinal and culinary properties. In recent decades, its multiple effects were proven, confirming its antirheumatic, anti-inflammatory, and gastroprotective properties, as well as in the treatment of bronchitis, flu, gastrointestinal ailments, and diuretics [7].

The phytochemical analyses of the extract of the genus *Sideritis* have shown various constituents [5, 8], such as terpenoids, coumarins, sterols, and especially flavonoid aglycones and glycosides. The chemical composition of *S. raeseri* has been reported earlier [7]. *Sideritis* constituents have been reported to exert various biological activities [5, 7, 9–12], including antitussive, stomachic, antimicrobial, anti-inflammatory, antinociceptive, carminative, antioxidant [10], vasoprotective, hypoglycemic, anti-HIV replication, antifeedant, antiulcer, anticataract, and immunomodulating functions, as well as hypotensive, vasorelaxant, and cardiodepressant activities [11].

We have previously reported a new tracheorelaxant effect of another medicinal plant, *Vitex agnus-castus* [13]. There are no prior studies that have revealed the mechanism of tracheorelaxant activity of *S. raeseri*. In view of the *S. raeseri* ethanol extract effects, we hypothesized that the *S. raeseri* extract may also have a tracheorelaxant function. This study was realized to scientifically demonstrate the pharmacological basis for medicinal use of the *S. raeseri* extract to be used for the treatment of hyperactive airway disorders, such as asthma, respectively.

## 2. Materials and Methods

### 2.1. Preparation of the Crude Plant Extract

The plant material (the shoot system) from SR was manually picked in Albania during 2018, free of any dust particles, and shade-dried away from strong winds. Subsequently, it was grinded to coarse powder by the grinding machine. The plant material was then soaked in 80% aqueous ethanol solvent for 24 h with occasional/continuous shaking. The material was passed through the filter paper. The filtrate was carefully concentrated by an evaporator under pressure. The dried extract was transferred into containers and kept in the refrigerator at 4°C for their future use. Different dilutions of the crude plant extract from the stock were made fresh on the day of the experiment.

### 2.2. Reagents

Acetylcholine chloride (ACh, induces smooth muscle (SM) contraction), carbachol (CCh, induces SM contraction), L-NAME (N^G^-nitro-ʟ-arginine methyl ester, an inhibitor of nitric oxide (NO) synthases (NOS)), bradykinin (stimulator of NOS), indomethacin (cyclooxygenase (COX) inhibitor), and ODQ (1H-[1, 2, 4] oxadiazolo[4, 3-a]quinoxalin-1-one, a selective inhibitor of nitric oxide-sensitive guanylyl cyclase) were purchased from Sigma-Aldrich, Germany. All other chemicals and reagents used for making specific physiological solutions and other analyses used in this study were of analytical grade. Bradykinin was dissolved in 0.1 M acetic acid concentration and ODQ in DMSO whereas indomethacin in ethanol (50 mg/mL). All other drugs were dissolved in distilled water unless otherwise stated. Worthy to mention, all the drugs were freshly made up and used on the day of the experiments.

### 2.3. Treatment and Sensitization of Rabbits

Adult rabbits (weighing 800–1200 g (gram)) of either sex were treated according to the law of animal welfare of the Republic of Kosova as well as according to good experimental practices. The protocol and procedures employed in this study were ethically reviewed and approved by the responsible bodies of Uni. Prishtina. All experiments were carried out in compliance with relevant national and international standards for animal experiments [14, 15], especially in compliance with the Directive 2010/63/EU. Rabbits were purchased from domestic suppliers, housed in standard conditions, 19°C–23°C and 12 h light/dark cycle, with food and water administered *ad libitum*. The rabbits were sacrificed following a blow on back of the head and dissected to extract the trachea for *in vitro* experiments with isolated tissues. The trachea was carefully cleaned of surrounding connective tissue while holding in the Krebs–Henseleit solution (KHS) [13]. The trachea was cut into small segments (2–3 mm) with 2 cartilage rings, each mounted between two stainless steel hooks within thermostatically controlled (37°C) organ baths. The lower hook was tied at the bottom of the organ bath, while the upper tissue hook was tied with an isometric force transducer (DMT 750, Danish Myo Technology, Denmark) connected to an ink-writing recorder. The mounted trachea segment was kept in an organ bath filled with a 10 mL volume of KHS (pH 7.4, 37°C) in the composition as follows (mM): NaCl (118), KCl (4.7), CaCl_2_ (2.52), MgSO_4_ (1.64), KH_2_PO_4_ (1.18), NaHCO_3_ (7), and glucose (5.5) and continuously bubbled with 5% CO_2_ and 95% O_2_.

### 2.4. Experimental Protocols

Isometric tension was continuously measured with a force transducer (DMT 750, Danish Myo Technology, Denmark). The trachea segments were manually stretched initially to 1 g initial tension, which was found to be optimal for measuring the changes in tension [11, 16, 17]. This passive tension was applied throughout the experiment, and subsequent changes in tension were recorded continuously using the PowerLab system with LabChart software (ADI Instruments, Denmark). Tracheal rings were allowed to equilibrate for 45–60 min before applying any test substance, during which the trachea ring segments were regularly washed out with KHS every 15 min, and resting tension 1 g was readjusted. After the equilibrium period, in an independent series of experiments, the trachea ring segments were preincubated (app. 5 min) without or with respective concentrations of the *S. raeseri* extract (0.5 or 1 or 2 mg/mL) and without (basal tonus (untreated)) or with ACh (0.3 nM–1 *μ*M) to induce tracheal smooth muscle contractions. In addition, in another set of experiments, other spasmogens, such as CCh (1 *μ*M) or KCl (80 mM), were applied on isolated trachea segments to induce smooth muscle constrictions, and when the plateau was achieved, the increased concentrations of the *S. raeseri* extract (0.3–2 mg/mL) were tested by their addition in a cumulative manner to the organ bath and left to achieve a new plateau for each indicated concentration. The control tracheal rings were measured in parallel with the experimental tissues.

### 2.5. Mechanistic Studies

The first step toward understanding how the *S. raeseri* extract alters airway responsiveness was to elucidate nitric oxide- (NO-) cyclic GMP (cGMP) [17, 18] as well as prostaglandin E2 (PGE2) [19], mediated mechanisms regulating tracheorelaxant activities induced by carbachol. The signaling mechanism by which NO induces tracheorelaxation is as follows: NO is produced in the endothelial cells and then diffuses into the adjacent smooth muscle cells, where it activates soluble guanylate cyclase leading to the production of the second messenger cGMP. This in turn stimulates muscle relaxation [17]. cGMP, in a process catalysed by PDE-5, is hydrolysed to inactive metabolite 5′GMP, thus ending the relaxation. Additional cellular signaling pathway involved in muscle relaxation is COX-1-PGE2 [19]. The synthesis of PGE2 from arachidonic acid is catalysed by COX.

To this end, to investigate the mechanisms of relaxation effects of the *S. raeseri* extract, the trachea rings were treated without or with CCh, without (CCh alone) or with 2 mg/mL of the *S. raeseri* extract, and without or with the inhibitor of NO synthases L-NAME (100 *μ*M), or stimulator of NOS bradykinin (100 nM), or selective inhibitor of guanylyl cyclase ODQ (10 *μ*M), or COX inhibitor indomethacin (10 *μ*M), respectively.

### 2.6. Statistical Analysis

Relaxant responses of the *S. raeseri* extract are expressed as a value of ACh-CCh-KCl-sensitive maximal trachea smooth muscle contractions compared to 1 g of contraction force. Data are expressed as means ± SEM and are representative of at least five repeated experiments for each series of experiments. Statistical analysis was made by ANOVA or *t*-test. A value of *p* < 0.05 was considered statistically significant.

## 3. Results

### 3.1. *S. raeseri* Extract Alone Has No Effect on the Tracheal Basal Tone

In the first series of experiments, we investigated the potential effect of the *S. raeseri* extract (SRE) alone on the tracheal basal tone after an approximately 1 h equilibration period under 1 g resting tension. To this end, the addition of cumulative concentrations of the *S. raeseri* extract (0.3–2 mg/mL) to the tissue bath did not significantly alter the tracheal basal tone (data not shown).

### 3.2. *S. raeseri* Extract Has Relaxant Function after Acetylcholine-Induced Contraction on the Trachea

**Figure 1:**
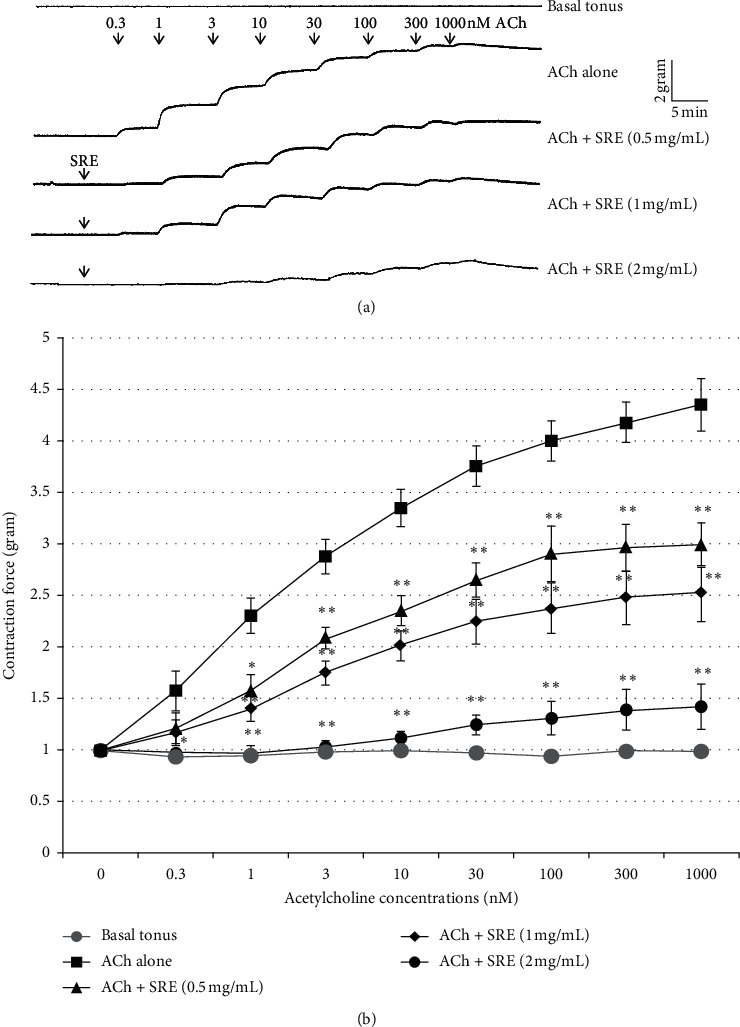
*S. raeseri* extract causes rabbit airway smooth muscle relaxation after muscle contraction induced by acetylcholine. (a) Original representative current tracings showing effects in force/time of dose-dependent (0.3–1000 nM) acetylcholine-induced smooth muscle contraction on isolated tracheal rings in the absence or presence of app. 5 min preincubation with specific concentrations (0.5, 1, and 2 mg/mL) of the *S. raeseri* extract. (b) Arithmetic means ± SEM (*n* = 5-6) of different measurements recorded in tracheal rings untreated (basal tonus, grey closed circles) or treated with specific dose-dependent concentrations of acetylcholine in the absence (closed squares) or after 5 min pretreatment in the presence of 0.5 mg/mL (closed triangles), 1 mg/mL (closed rhombi), or 2 mg/mL (black closed circles) of the *S. raeseri* extract. ^∗^indicates statistical significance (*p* < 0.05; *t*-test) from the absence of the *S. raeseri* extract. ^∗∗^indicates significance (*p* < 0.01; *t*-test) from the absence of the *S. raeseri* extract.

Pretreatments of rabbit tracheal rings with the ethanol *S. raeseri* extract (0.5 mg/mL) resulted in a significant relaxation of the muscle after an increased concentration of acetylcholine (0.3–1000 nM) contractile stimulus compared to acetylcholine alone (Figure 1). Increased concentrations (1 or 2 mg/mL) of the *S. raeseri* extract further potentiated the relaxant effects of the extract, return of the concentration-response curve toward baseline, respectively. This significant (*p* < 0.01) inhibition of smooth muscle contraction by the SR extract clearly indicates *S. raeseri* extract anticholinergic activity.

### 3.3. The Tracheorelaxant Effect of the *S. raeseri* Extract after Carbachol and High K^+^-Induced Contraction

**Figure 2:**
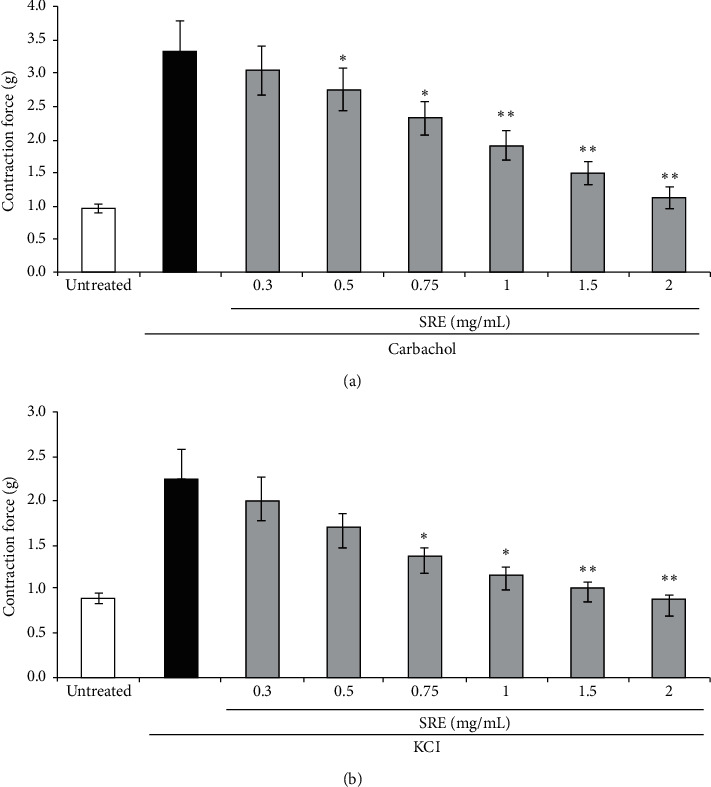
The dose-dependent relaxant effect of the *S. raeseri* extract on CCh- and KCl-induced tracheal ring smooth muscle contractions. Arithmetic means ± SEM (*n* = 5-6 segments, each taken from a different rabbit) of the tracheorelaxant function of the *S. raeseri* extract (0.3–2 mg/mL, grey bars) on tracheal muscle contraction induced by CCh (1 *μ*M) (a) and KCl (80 mM) (b). Results are expressed relative to values obtained at 1 g of contraction response. ^∗^denotes statistical significance (*p* < 0.05; *t*-test) from the absence of the *S. raeseri* extract. ^∗^^∗^indicates significance (*p* < 0.01; *t*-test) from the absence of the *S. raeseri* extract.

In the next series of experiments, the tracheal rings were treated with *S. raeseri* extract after their muscle was precontracted by carbachol (also known carbamylcholine, CCh), high (80 mM) K^+^, respectively (Figure 2). The cumulative concentration of the *S. raeseri* extract (0.3–2 mg/mL) significantly reduced high CCh- and K^+^-evoked tracheal contraction in a concentration-dependent manner. Specifically, the cotreatment of rabbit tracheal rings with CCh and *S. raeseri* extract resulted in significant decrease in the muscle contraction effect in rabbits as compared with CCh alone, reaching the statistical significance at 0.5 mg/mL of the *S. raeseri* extract (*n* = 5) (Figure 2(a)). The *S. raeseri* extract-induced relaxant effect was more pronounced in higher *S. raeseri* extract concentrations precontracted with CCh.

Similarly, *S. raeseri* extract also showed its relaxant effect against high K^+^-induced contractions (80 mM) in rabbit tracheal preparations (Figure 2(b)). The *S. raeseri* extract caused inhibitory effects in a cumulative dose-dependent manner (0.3–2 mg/mL). As presented in Figure 2(b), pretreatment of the *S. raeseri* extract at the doses of 0.75 and 1 mg/mL significantly (*p* < 0.05, *n* = 5-6) relaxed the muscle, whereas the effect was more potentiated at 1.5 and 2 mg/mL (*p* < 0.01) as compared with high K^+^ alone; therefore, *S. raeseri* extract clearly exerted its relaxant effects in CCh as well as high K^+^-induced trachea muscle contraction. However, the *S. raeseri* extract-mediated tracheorelaxant effect was more pronounced in CCh-induced airway smooth muscle (ASM) contraction than in high K^+^.

### 3.4. Role of NO/cGMP Production in *S. raeseri* Extract-Induced Tracheorelaxant Effect

**Figure 3:**
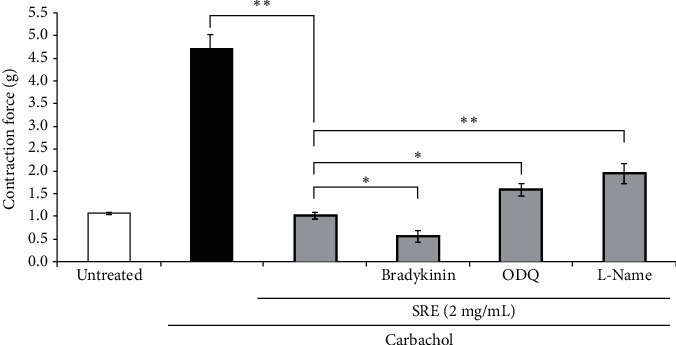
Bradykinin potentiated, whereas L-NAME and ODQ inhibited the relaxant effects of the *S. raeseri* extract precontracted with CCh. Arithmetic means ± SEM (*n* = 6-7) of the untreated (1^st^ bar) and the relaxation responses precontracted with CCh (1 *μ*M, 2^nd^, 3^rd^, 4^th^, 5^th^, and 6^th^ bars) without (2^nd^ bar) or with (3^rd^, 4^th^, 5^th^, and 6^th^ bars) the *S. raeseri* extract (2 mg/mL) and without (3^rd^ bar) and with (4^th^ bar) additional preincubation with 100 nM bradykinin, 10 *μ*M ODQ (5^th^ bar), and 100 *μ*M L-NAME (6^th^ bar), respectively, for 5 min. ^∗^shows statistical significance (*p* < 0.05; *t*-test); ^∗^^∗^denotes significance (*p* < 0.01; *t*-test) between treatments as shown in the figure.

Another series of experiments were realized to examine the potential role of NO/cGMP-mediated signaling in the tracheorelaxant effect of the *S. raeseri* extract. To this end, tracheorelaxant function of the *S. raeseri* extract (2 mg/mL) on CCh-induced tracheal ring contraction was further potentiated after incubation with bradykinin (NOS stimulator, 100 nM) (*p* < 0.05, *n* = 5-6), whereas ODQ (inhibitor of soluble guanylyl cyclase, 10 nM) (*p* < 0.05, *n* = 5) or L-NAME (nonspecific NOS inhibitor, 100 *μ*M) significantly decreased the *S. raeseri* extract-induced relaxation (*p* < 0.01, *n* = 5) in the isolated rabbit trachea preparations compared with *S. raeseri* extract (and cCCh) treated alone (Figure 3).

### 3.5. *S. raeseri* Extract-Induced Relaxant Activity Is Also Associated with Prostaglandin E2

**Figure 4:**
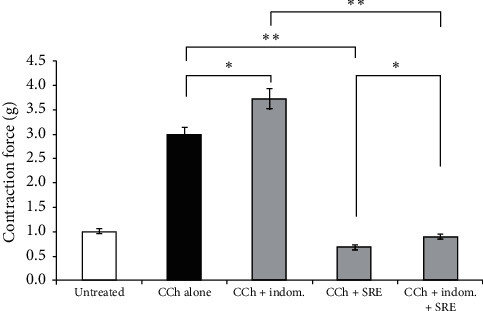
Indomethacin decreased the relaxant effects of the *S. raeseri* extract precontracted with CCh. Arithmetic means ± SEM (*n* = 9-10) of the basal tonus (1^st^ bar) and the relaxation responses precontracted with CCh (1 *μ*M) without (2^nd^ and 3^rd^ bars) or with (4^th^ and 5^th^ bars) the *S. raeseri* extract (2 mg/mL) and without (1^st^, 2^nd^, and 4^th^ bars) and with (3^rd^ and 5^th^ bars) additional preincubation with 10 *μ*M indomethacin for 5 min. ^∗^denotes statistical significance (*p* < 0.05; *t*-test) and ^∗^^∗^indicates significance (*p* < 0.01; *t*-test) which are presented within the figure.

One of the mechanisms involved in smooth muscle relaxation is PGE2 production, a product of COX-1 activation [19]. To ascertain whether, or not, the PGE2 signaling pathway is involved in *S. raeseri* extract-dependent tracheorelaxant activity, indomethacin (nonselective COX inhibitor, 10 *μ*M) was added prior to 2 mg/mL of the *S. raeseri* extract in CCh-contracted tracheal rings. Compared to *S. raeseri* extract-treated alone (in CCh precontracted muscle), the cotreatment of rabbit smooth muscles with the *S. raeseri* extract and indomethacin significantly inhibited the *S. raeseri* extract-induced relaxant effects (Figure 4) (*p* < 0.05, *n* = 5).

## 4. Discussion

This study demonstrates that the *S. raeseri* extract has relaxant effects following smooth muscle-induced contractions by distinct spasmogens, including ACh, CCh, and high K^+^ stimuli. Thus, suggesting *S. raeseri* extract has anticholinergic activity, involving Ca^2+^ entry blockade. Furthermore, the SRE-dependent relaxant effect was decreased by the exposure to the NOS inhibitor, L-NAME, by the guanylyl cyclase inhibitor, ODQ, and by the COX inhibitor, indomethacin, whereas it was increased by the NOS stimulator, bradykinin. This shows that both pathways, NO/cGMP and PGE2, contribute to the SRE-sensitive tracheal relaxations.

In addition to the previously reported hypotensive, vasorelaxant, cardiodepressant [11], and antioxidant effects [10], the *S. raeseri* extract showed tracheorelaxant activity. Specifically, our study demonstrated that the ethanol extract of *S. raeseri* possesses a tracheorelaxant effect against increasing concentrations of ACh (0.3 nM to 1 *μ*M). SRE could act directly via cholinergic receptors localized on the cells of the tracheal smooth muscle [20] and exert its effects through cholinergic mechanisms, possibly mediated by calcium channel blockade [20–23].

The extract of *S*. *raeseri* also exhibited a dose-dependent tracheorelaxant effect when tested on rabbit tracheal rings precontracted with both CCh and high K^+^. CCh- and high KCl-induced contractions are associated with membrane depolarization, leading to an increase in the Ca^2+^ influx across the plasma membrane through voltage-dependent channels [23–25]. The tracheal smooth muscle contractions are initiated by cholinergic agonists such as CCh and high K^+^ through stimulation of muscarinic receptors that lead to the opening of L-type Ca^2+^ channels, and consequently enhancing intracellular calcium levels ([Ca^2+^]_i_), therefore, resulting in tracheoconstriction [21, 24, 26]. Compared to high K^+^, which leads to muscle contraction by allowing the influx of Ca^2+^ from the extracellular space into the cytosol through the cell membrane, CCh induces contraction by two pathways, either one leading to influx of external Ca^2+^ in the cytosol and/or another releasing Ca^2+^ from intracellular stores, sarcoplasmic reticulum [22, 23, 27]. Accordingly, the SRE appears to involve these two pathways. Our results are entirely consistent with the findings of the previous study reporting the SRE vasorelaxant effect [11]. Nonspecific inhibition of both CCh and high K^+^ by the SR extract indicates that nonspecific tracheorelaxant function, apparently, at least partly is accomplished by calcium channel blockade mechanisms. Similar tracheorelaxant effect was reported in our recent research, but for another medicinal plant species, *Vitex agnus-castus* [13].

The NO/cGMP pathway induces smooth muscle relaxation through multiple mechanisms [28]. NO is an endogenous tracheorelaxant mediator that is made in a reaction catalysed by the NO synthase (NOS) isoforms, which utilize L-arginine, molecular oxygen, and NADPH as substrates to effectively produce NO and L-citrulline [29, 30]. Nitric oxide further activates soluble guanylyl cyclase (GC) to synthesize intracellular cGMP, relaxing the airway smooth muscle [18]. We preincubated isolated tracheal rings with either NOS inhibitor L-NAME or NOS stimulator bradykinin or GC inhibitor ODQ to determine the putative involvement of the NO/cGMP-mediated signaling pathway in the relaxant activity of *S. raeseri*. Our findings revealed that treatment with L-NAME and ODQ decreases or bradykinin increases the tracheorelaxant activity of SRE. Therefore, it clearly indicates that SRE-induced tracheorelaxant effects are mediated by multiple mechanisms, including NO/cGMP signaling pathway.

Nonetheless, an additional pathway (i.e., COX-1-PGE2-mediated relaxation [19]) appears to participate in SRE-induced smooth muscle relaxation in rabbit tracheal rings. SRE significantly declined, whereas COX inhibitor indomethacin enhanced the tracheal smooth muscle constructive effect precontracted with CCh. In this line, pretreatment with indomethacin declined the relaxant effect of the SRE in tracheal rings precontracted with CCh. However, the prostaglandin E2 pathway seems to be involved in the action of the SRE, although to a lesser extent compared to the NO/cGMP pathway.

## 5. Conclusions

These findings suggest that the SRE possesses airway-relaxing properties that are mediated by NO/cGMP- and COX-1-PGE2-dependent mechanisms and inhibition of calcium entry, respectively. Taken together, our study data recommend SRE as a newly available treatment for limited airflow resulting from many lung diseases such as asthma and other respiratory manifestations.

## Data Availability

The data results used to support the findings of this study are included within the article, while the database is deposited in our laboratory (Fac. of Medicine, Uni. Prishtina).
